# Current Knowledge of Silver and Gold Nanoparticles in Laboratory Research—Application, Toxicity, Cellular Uptake

**DOI:** 10.3390/nano11092454

**Published:** 2021-09-21

**Authors:** Patrycja Talarska, Maciej Boruczkowski, Jakub Żurawski

**Affiliations:** Department of Immunobiology, Poznan University of Medical Sciences, 60-806 Poznań, Poland; maciej.boruczkowski@gmail.com

**Keywords:** silver nanoparticles, gold nanoparticles, nanotoxicology, nanoparticle interactions, nanoparticle applications

## Abstract

Silver and gold nanoparticles can be found in a range of household products related to almost every area of life, including patches, bandages, paints, sportswear, personal care products, food storage equipment, cosmetics, disinfectants, etc. Their confirmed ability to enter the organism through respiratory and digestive systems, skin, and crossing the blood–brain barrier raises questions of their potential effect on cell function. Therefore, this manuscript aimed to summarize recent reports concerning the influence of variables such as size, shape, concentration, type of coating, or incubation time, on effects of gold and silver nanoparticles on cultured cell lines. Due to the increasingly common use of AgNP and AuNP in multiple branches of the industry, further studies on the effects of nanoparticles on different types of cells and the general natural environment are needed to enable their long-term use. However, some environmentally friendly solutions to chemically synthesized nanoparticles are also investigated, such as plant-based synthesis methods.

## 1. Introduction

Nanoparticles are defined as structures with at least one of the dimensions in the 1 to 100 nm range [1]. These particles enter cells mostly through endocytosis, particularly endocytotic vesicles formation and the release of ions into the cytoplasm [2,3,4].

From the clinical standpoint, the use of nanoparticles (NPs) is mainly motivated by their relatively large surface-to-volume area during interaction with cells. Further advantages include their specific physicochemical characteristics, such as catalytic properties and relatively low melting point (compared to the macroscopic properties of the metal they are derived from). Moreover, to ensure the safety of their use and appropriate dosage, correlations between these characteristics and the potential toxicity of nanoparticles can be determined using nanotoxicology techniques [5,6,7].

NPs can be characterized according to their shapes. These include simple spherical, triangular, rod, triangle, and round, and more complex octagonal or polyhedral [4,8,9]. Gold nanoparticles can come in a variety of shapes including nanorod, nanostar, nanosphere, nanocube, nanoshell, nanocluster, suboctahedral, icosahedral tetrahedral, decahedral, and oroctahedral [10]. Silver nanoparticles also exhibit different shapes including spherical, nanorod, nanowire, nanobar, nanoplatele, triangles, five or six diagonal, cubic, and pyramid [11]. The shape and size of nanoparticles affect their use in various industries, as these properties reflect their optical, electronic, magnetic, and catalytic characteristics [11,12].

It has been proven that silver nanoparticles (AgNP) have the ability to penetrate the cellular walls of bacteria, altering their cell membranes and even potentially causing cell death. Moreover, through the release of silver ions, it is possible to increase cell membrane permeability, produce reactive oxygen species, and disturb DNA replication [13,14,15,16].

Gold nanoparticles (AuNP) exhibit significant biocompatibility, promote corrosion, and possess optical and electronic properties depending on their shape and size [16,17,18]. AgNP also exhibit optical and electronic properties dependent on size, shape, surface coverage, and agglomeration [19,20,21]. Due to the optical properties of silver nanoparticles, they strongly interact with specific wavelengths of light, to which they have found wide use in biomedical applications, e.g., in vitro cellular imaging systems [21]. Due to their optical properties, noble metal nanoparticles can be used, for example, as an active ingredient in SPR (surface plasmon resonance) biosensors [22,23,24,25].

## 2. Nanoparticle Applications

The rapid development of nanotechnology is constantly affecting the methods of diagnosis, prevention, and treatment of various diseases, enabling novel therapeutic approaches. NPs currently have a range of applications, including, e.g., antibacterial agents, and components of drug delivery systems and diagnostic tools. It is worth noting that they are also commonly used as components of skincare products and cosmetics [26,27].

AgNPs are among the most commonly used substances in consumer products, such as laundry machines, dusting cloths, and personal hygiene products. Hence, NPs contained in everyday household items are often discarded directly in sewage and can potentially be transported into waterways. They are mainly described to exhibit antibacterial properties [28,29,30,31]. Typical AgNP applications include coatings of cloth and other textiles, food storage appliances, and cosmetics. They are also present in various applications used in the public health sector, and in medical products, such as disinfectants, wound dressings, central venous caterers, and surgical nettings. Furthermore, study results indicate that AgNPs may also exhibit cytotoxic properties, as they induce a typical cellular reaction of reactive oxygen species (ROS) formation [12,16,32,33,34]. AgNPs have also shown antibacterial (against *Staphylococcus aureus, Pseudomonas aeruginosa*, *Xanthomonas axonopodis pv. Citri*), fungicidal (against *Candida albicans, Candida parapsilosis*), and antiviral properties, also affecting the SARS-CoV-2 virus [35,36,37]. In addition, the AgNPs toxic effect, in the range of 50% of lethal concentration (LC 50) on certain young and adult fish species (zebrafish), daphnia, and at least two algae species, has been described [38,39]. Silver in ionic form (Ag^+^) is also known to be toxic to aquatic organisms even at g/L concentrations by inhibiting the effects of ATPase Na^+^/K^+^ and causing ionic imbalance. These effects of AgNPs were similar in different marine animals, such as fish, daphnia, and crayfish [40,41].

AgNPs are easily assimilated by cells. Therefore, they are mainly applied in the biomedical industry due to their unique surface, electronic, and optical properties. They are also used as innovative tools in diagnostic research, e.g., the detection of heart disease or cancer biomarkers, and in drug delivery systems [16,42,43,44,45,46,47]. Numerous reports have been published regarding the toxicity of gold nanoparticles depending on their size, shape, or structure of the coating and the spectrum of experimentally measured parameters. Toxicity depending on the size of gold nanoparticles in relation to different cell types has been described, demonstrating that smaller nanoparticles are generally more toxic [48,49].

In contrast, other teams of scientists have found no cytotoxic effects in cancerous cell lines treated with gold nanoparticles [25,50,51]. Subsequent works noted adverse effects on cytoskeleton components and a decrease in the growth of human cells exposed to AgNPs under culture conditions. Furthermore, increased levels of ROS were reported in aquatic organisms exposed to gold nanoparticles [52,53,54,55,56].

## 3. Absorption of Silver Nanoparticles

Silver nanoparticles can be absorbed through the respiratory and digestive systems, and through the skin [33,57].

In Sprague–Dawley rats subject to inhalation exposure, silver nanoparticles were detected in the blood and lungs. In smaller amounts, they were also seen in other internal organs such as the liver, kidneys, spleen, heart, and brain. These experiments did not describe changes in body weight gain, internal organ weight, or biochemical parameters. However, there were some differences in respiratory parameters in rats, e.g., decreased respiratory volume and minute ventilation, and histopathological changes in the lungs at 90-day exposure to 20 nm silver nanoparticles at 49, 133, and 515 µg/m^3^ doses [55]. In Sprague–Dawley rats subjected to 28-day exposure to AgNP aerosol, the appearance of multinucleated macrophages was observed in the lungs, which indicates inflammation and active absorption of nanoparticles at the dose of 30.5 μg/m^3^ [58].

Spherical AgNP (20 nm) at doses of 50, 150, 300 mg/kg can be ingested orally, absorbed through the intestines into the bloodstream, and then accumulate in other organs such as the duodenum, liver, kidneys, and spleen. While this has been confirmed in mice, the authors of the study did not report any associated pathological changes [59]. Moreover, mice subjected to 14-day oral AgNP administration (dose: 1 mg/kg, size: 22, 42, 71, and 323 nm) also showed no histopathological changes in the liver, kidneys, testicles, or lungs. After 28 days and only in a dose of 1 mg/kg (size: 42 nm), only a small cellular infiltration was observed in the renal cortex. However, based on an increase in pro-inflammatory cytokines, induction of inflammation by AgNP incubation was confirmed. Furthermore, there was also an increase in liver enzymes [60].

In addition, Guinea pigs were found to accumulate free aggregate AgNP in the epidermis layers after 24 h of exposure at the highest dose used (100,000 ppm). However, microscopic evaluation did not reveal any abnormalities in the epidermis and skin layers in exposed areas of groups treated with colloidal AgNPs (spherical or polygonal shape, size: 10–20 nm) compared to controls [61]. The permeability of silver nanoparticles from textiles was also investigated. In the study by Bianco et al. (2016), volunteers wore an AgNP-containing sleeve on their forearm (average concentration 3.6 *w/w*), 8 h at night for five consecutive days. While the study confirmed the presence of AgNP aggregates in the skin, deeper layers there were less affected than those closer to the surface [62].

Previously, George et al. (2014) demonstrated the presence of clusters of silver in the stratum corneum and deeper layers of the epidermis after five days of skin exposure of healthy individuals to nanocrystal silver dressing (amount of silver released by dressing: 70 ppm of silver ions, size of particles: 10–40 nm). Thus, they confirmed the possibility of AgNP penetration through intact skin. However, despite the deposition of silver in the dermis, silver nanoparticles did not reach systemic circulation and should therefore not have systemic consequences [63].

## 4. Absorption of Gold Nanoparticles

Studies in rats show that gold nanoparticles can be absorbed through the respiratory and digestive systems [33,64].

In Sprague–Dawley rats subjected to spheroid AuNP (diameter under 6 nm) inhalation for 90 days, a decrease in respiratory parameters, i.e., lung function, respiratory volume, and minute volume, was observed compared to the control. Furthermore, histopathological examination demonstrated minimal alveoli, inflammatory infiltration of mixed cell type (lymphocytes/neutrophils/macrophages), and increased macrophage counts in rats receiving high doses of AuNP (20 μg/m^3^) [64].

Moreover, studies by Kreyling et al. (2018) also confirmed the absorption of “potato-shaped” gold nanoparticles (size: 20 nm, density: 19.5 g/cm^3^) by inhalation in rats. About 30% of AuNP accumulated in the epithelium of the respiratory tract, causing rapid mucociliary removal and swallowing into the gastrointestinal system. Long-term removal (after 28 days) of AuNP was dominated by macrophage-mediated transport through interstitial tissue to the larynx and gastrointestinal tract. Furthermore, AuNP retention has also been observed in the liver, spleen, kidneys, uterus, and brain [65].

In Wistar rats, ten days after intravenous administration of 25 nm colloidal AuNPs (0.3619 mg of particles/mL, per 1 kg), more than 50% of AuNP accumulated in the liver with smaller amounts in the lungs and spleen. This occurrence was associated with the collection of AuNPs from the circulation by the mononuclear phagocyte system. The total AuNP content of all organs represented 60% of the initial dose. In contrast, oral administration showed almost 50 times lower AuNP levels at the same amount (1.4% of the initial dose). Most AuNP was excreted in feces within four days after exposure. In turn, alterations in biochemical parameters were observed 72 h after intravenous AuNP administration. An increase in AST (aspartate aminotransferase) was observed, with a decrease in ALT (alanine aminotransferase), which affects the physiology of the liver. Furthermore, an increase in blood glucose has also been noted; thus, the effect of AuNP on pancreatic functions cannot be excluded [66].

The penetration of gold nanoparticles through the skin of the hind paw and the anterior abdominal wall of Sprague–Dawley rats was also confirmed by Raju et al. (2018), with smaller AuNPs (22 nm) showing higher penetration compared to larger nanoparticles (105 and 186 nm). The effect of 3-hour AuNP incubation on a fibroblast cell line (L929 mouse fibroblast cells) was also investigated, with no observed effect of AuNP on cell viability at any of the concentrations used (0.1, 1, and 10% *v/v*) [67].

In mice, the kidneys were the primary site of AuNP accumulation after oral administration for 8 days at 25, 22, 20, 18, and 15 μg gold/kg bodyweight concentrations, and subsequent intestinal absorption. AuNP can induce anti-inflammatory effects in macrophage RAW264.7 cells pretreated with 1/1000 OD of the AuNP for 5 h before stimulation with lipopolysaccharides (LPS) and incubation for another 20 h. AuNP reduce by reducing the lipopolysaccharide receptor expression on the cell surface, as well as catalytic detoxification of nitrite peroxide and hydrogen peroxide. The highest accumulation of gold nanoparticles was shown by those that were 5 nm in size and coated with PVP, compared to 5 nm AuNPs coated with citrate or tannic acid (TA) [68].

In other studies conducted in men subjected to nanoparticle inhalation for 2 h during intermittent exercise, AuNP was also confirmed to enter the lungs. Gold was detected in the urine after exposure to 4 nm AuNPs, but not in the urine of volunteers exposed to larger particles (34 nm). In mice, gold nanoparticles were also detected in urine only after exposure to particles ≤5 nm. AuNP found in human blood was usually at low levels after inhalation of AuNP, although the concentration of smaller particles was notably higher. This effect was also confirmed in mice, in which the incidence of detected gold and blood gold levels were significantly higher after exposure to smaller particles [69].

The possibility of absorption through human skin has been studied using surgically resected dermal fragments incubated for 24 h with AuNP. The permeability of spherical nanoparticles (15 and 100 nm) was confirmed by using a TEM (transmission electron microscope), with nanoparticles observed in the deeper stratum corneum, epidermis, and dermis [70].

## 5. Nanoparticle Toxicity

Excessive ROS production can cause DNA damage and activate several signaling pathways, i.e., p53 suppressor protein, AKT (serine/threonine protein kinase B), and MAPK (mitogen-activated protein kinases) [71]. Furthermore, nanoparticle toxicity may increase pro-inflammatory cytokine expression and activation of pro-inflammatory cells such as macrophages and neutrophils, which result in increased ROS production [2].

In various physiological states, ROS are produced as intermediate products. Their concentrations in cellular organelles are strongly regulated by various detoxifying enzymes, such as superoxide dismutase (SOD), glutathione peroxidase (GPx), and catalase (CAT), or by different antioxidants including flavonoids, ascorbic acids, vitamin E, and glutathione (GSH). The production of free radicals induced by nanoparticles leads to a reduction of GSH to oxidized form, followed by induction of oxidative stress [2,12,72,73,74,75].

### 5.1. Silver Nanoparticle Toxicity

Based on the data shown in Table 1, it appears that an increase in AgNP levels affects the change in cell morphology and viability, and the production of ROS [76,77,78,79,80,81,82,83,84].

A more significant impact of smaller nanoparticles on increased apoptosis induction, and an increase in dye fluorescence intensity resulting from increased production of reactive oxygen species compared to larger nanoparticles, were also observed [85,86,87,89]. In the cited works [86,87,89], monitoring of reactive oxygen species formation in the material studied was investigated by measuring the DCFH-DA fluorescence intensity. In turn, in the publication by Sriram [85], the level of ROS production was determined through nitrotetrazolium blue (NBT) reduction assay. Rat alveolar macrophages showed higher fluorescence intensity of the DCFH-DA dye with smaller size of silver nanoparticles (15 nm), compared to 30 and 50 nm. There was also an increase in dye fluorescence intensity with increasing concentration of silver nanoparticles (5, 10, 25, 50, 75 μg/mL) [89]. Studies on human lymphocytes showed a correlation between the increasing fluorescence intensity of the DCFH-DA dye and concentrations of silver nanoparticles (10, 20, 75, 100 μg/mL) [86]. HepG2 cells showed the most significant increase in DCFH-DA dye fluorescence in the presence of the smallest nanoparticles (5 nm) compared with 20 and 50 nm [87].

A link between the phenomenon of apoptosis and incubation of silver nanoparticles in different sizes has been proposed by researchers:

Carlson et al. [89]—Based on the intensity analysis of fluorescent cationic dye, 5,5′,6,6′-tetrachlor-1,1′,3,3′- tetraethyl-benzamidazolocarbocyanine iodide (JC-1), it was shown that 55 nm AgNPs, in contrast to 15 and 30 nm, did not lead to significant toxicity at concentrations up to 50–75 μg/mL. The authors suggest that the loss of mitochondrial membrane potential (MMP) function may be due to apoptosis arising from the mitochondrial apoptotic pathway.

Liu et al. [87]—The study investigated median effective concentration for cell mortality (EC50) values, based on both mass concentration and surface area, in A549, HepG2, MCF-7, and SGC-7901 cell lines. It was shown that silver nanoparticles with the smallest size (5 nm) were the most toxic material to cells, compared to 20 and 50 nm.

Sriram [85]—Analysis of the effects of AgNP concentrations ranging from 100 to 1000 nM on BREC cells showed that the smaller ones (22.4 nm) induced apoptosis at concentrations of 300 nM and above, while the larger ones (49.5 nm) at concentrations above 500 nM.

Smaller nanoparticles cause structural modifications, e.g., changes in lymphocyte cell membrane morphology with nanoparticles ≤ 20 nm, with no similar effects observed for particles ≥ 200 nm in size [86]. Cell shrinkage and no visible plasma membrane were observed in rat alveolar macrophages after treatment with 15 nm, but not 30 nm AgNPs [89]. Furthermore, larger AgNPs (42.5 nm) did not induce cell shrinkage in BREC cells, unlike those of smaller sizes (22.4 nm) [85]. In HepG2 cells, swelling occurred at the smallest AgNP size (5 nm), while when incubated with larger NPs (20 and 50 nm), some cells retained their typical structure. In contrast, Hoechst 33342 analysis showed condensation of cell nuclei at AgNP size of 5 nm, while most nuclei of cells incubated with AgNPs of 20 and 50 nm were standard [87]. It was also noted that changing the external molecular properties of NPs through reactive groups on their surface modifies their effects on cellular processes [2], with some nanoparticles able to form aggregates or agglomerates. In rat alveolar macrophages, cells incubated with larger nanoparticles (30 nm) showed agglomeration of nanoparticles both inside and outside, while at 50 nm, agglomeration occurred only on the cell surface [89]. Structural modifications and changes in external molecular properties, which ultimately leads to the formation of reactive groups on the particle surface [2], which can directly result in ROS production. In addition, the adsorption of surrounding particulate matter, such as ozone and nitric oxide, on the NP surface affects the induction of oxidative stress [2,90]. Small NPs appear to be more toxic than large NPs, which may be explained by a relatively larger surface area to volume ratio compared to larger NPs [30].

With a low concentration of nanoparticles, cells are capable of defending against oxidative stress through antioxidant action, restoring redox balance. However, this mode of action is not possible at higher concentrations, causing toxicity of cells and inflammation [2,12,75,76,77,78,81,82,83,84,85].

**Figure 1:**
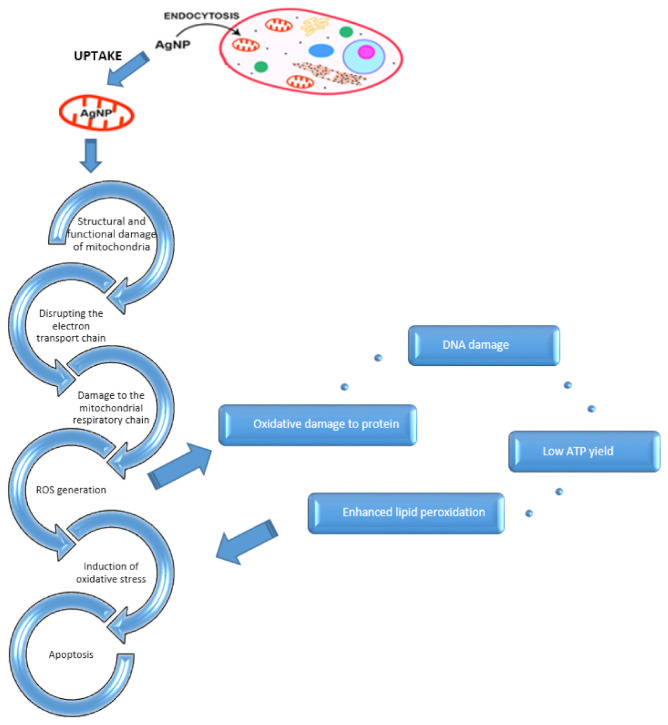
A possible mechanism of apoptosis induced by AgNP.

An increase in the toxicity of nanoparticles associated with the production of reactive oxygen species has also been described [76,77,79,80,83,91]. After infiltrating the cell, Ag nanoparticles degrade intracellularly, releasing Ag^+^ ions that impair mitochondrial function. ROS, accumulating as byproducts of the electron transport chain, cause mitochondrial damage and impairment of function, depolarization of the mitochondrial membrane, damage to mtDNA (mitochondrial DNA), and peroxidation of lipids and protein elements, eventually leading to apoptosis (Figure 1) [76,77,78,79,80,81,83,84,85,92].

The initial NAC (N-acetyl-L-cysteine) treatment of human Chang liver cells [78], human liver cancer cell line (HepG2) [80], human lung cancer cell line (A549) [82], and mouse line of DC2.4 dendritic cells [93] reduced ROS production in cells incubated with AgNPs. The use of synthetic NAC antioxidant in cells incubated with AgNP resulted from a change in mitochondrial membrane permeability, preventing the loss of its potential, which is a characteristic feature of apoptosis induction [93].

The use of instrumental neutron activation analysis (INAA) by Antsiferova et al. (2015) allowed for studying biokinetics of silver nanoparticles in biological tissues. The results confirm the highest uptake of nanoparticles through the liver and blood by mice, with a single exposure to 34 nm AgNP, compared to the brain. The animals were incubated with AuNP at a concentration of 100 μg/mL for one day. Furthermore, with an increase in incubation time of up to two months and the same dose, a higher accumulation of nanoparticles was observed in the liver than in the blood. The effectiveness of a month-long distilled water feeding on the level of silver nanoparticle removal from the organism after two months of exposure was also investigated, with the fastest reduction in AuNP observed in the liver. The study confirmed the ability of the liver and blood to quickly dispose of silver nanoparticles. It also ruled out the possibility of dangerous effects of AgNP on these organs [94].

The research Jo et al. (2020), conducted on Sprague–Dawley rats exposed for 28 days to silver nanoparticle aerosols of variable size (18.1–19.6 nm) and three concentration groups: small (31.2 ± 8.5 μg/m^3^), medium (81.8 ± 11.4 μg/m^3^), and high (115.6 ± 30.5 μg/m^3^), confirmed the presence of AgNPs in bronchoalveolar lavage (BAL). Furthermore, similar to previously mentioned studies, no effect of nanoparticles on body weight was noted. In turn, there was a statistically significant change in the weight of the right lung after a 7-day post-exposure observation based on average concentration. Biochemical changes, such as hemoglobin concentrations in erythrocytes, lymphocyte percentage, asparagine transaminase (AST), and lactate dehydrogenase (LDH) levels, were also observed at each of the concentrations used. Based on the number of neutrophils, indicating inflammation, significant increases were observed in high concentration groups [58].

### 5.2. Gold Nanoparticle Toxicity

Kadhim et al. (2021) investigated the effects of spherical gold nanoparticle toxicity on the in vitro rat model of embryonic fibroblast cells (REF), and in vivo—male mice weighing 25 to 30 g. REF cells were incubated for 48 h with AuNP concentrations of 1, 5, and 10 μg/mL. In turn, gold nanoparticles were intraperitoneally administered in mice, at a dose of 100 μg/Kg, for 28 days. Toxicity in REF cells was investigated using an MTT test. Furthermore, histopathological analysis of preparations from the liver, lungs, and spleen of mice was carried out using a fluorescent microscope. REF cells showed toxicity only at 10% at the highest AuNP dose. For concentrations of 1 and 5 μg/mL, this parameter was even lower. The analyzed cells showed no change in nuclear morphology, and no changes in histopathological slices. Moreover, nanoparticles did not affect the bodyweight of mice. The results of these in vitro and in vivo tests therefore confirmed the safety of gold nanoparticle use at low concentrations [45].

The effects of spherical AuPEG toxicity in mice were also studied by Chinese researchers in 2011. The study focused mainly on the size of nanoparticles, with four different sizes used: 5, 10, 30, and 60 nm. Mice were treated with gold nanoparticles at a concentration of 4000 μg/kg bodyweight for 28 days. A particle analyzer was used to assess the concentration of nanoparticles in the heart, lungs, spleen, and kidneys. Using a transmission electron microscope, preparations of bone marrow and blood were also characterized. Furthermore, biochemical blood tests were performed to evaluate the numbers of morphotic elements and enzyme levels.

Both the heart and kidneys exhibited the highest concentration of 5 nm nanoparticles. In turn, 10 nm nanoparticles were most commonly detected in the liver, while those 30 nm mainly aggregated in the spleen. Five nanometer nanoparticles were also observed in the bone marrow without any significant reduction in their size, indicating that they did not undergo disintegration. Furthermore, other nanoparticle sizes were also localized in both intracellular and extracellular compartments of bone marrow, indicating longer NP retention in this tissue.

In blood, nanoparticles of 5 nm were aggregated and could form 10–20 nm long structures. A similar situation was observed at 10 and 60 nm but not at 30 nm. Moreover, the presence of gold nanoparticles after 28 days of incubation indicated their long retention time in blood.

Due to the lack of immune response caused by gold nanoparticles of different sizes, no statistically significant differences of the mg/g index were detected between the study and control groups in either the thymus or the spleen.

Hematology results after 28 days of intraperitoneal injection at the dose of 4000 μg/kg gold nanoparticles are shown in Table 2.

An increase in the number of white blood cells observed in mice treated with 10 nm particles indicates an inflammatory reaction. In contrast, a decrease in the number of white blood cells observed in mice treated with 5 and 30 nm particles may be associated with infection. Furthermore, the increase in the number of red blood cells found in mice treated with 10 and 60 nm nanoparticles indicates that particles of this size affect the hematopoietic system.

The level of biochemical enzymes in the blood of mice was also investigated (Table 3).

Biochemical changes suggest that 10 nm particles may be highly toxic to the liver, and 60 nm particles could present toxicity to both the kidneys and liver. However, no liver and kidney damage was observed for 5 and 30 nm AuPEGs.

The study results suggest that 10 nm gold nanoparticles coated with PEG are not sufficiently safe to be administered at a concentration of 4000 μg/kg. The results of the studies presented above contradict previous assumptions linking in vitro cell incubations with smaller nanoparticles with higher toxicity [95].

Lasagna-Reeves et al. (2010) investigated the possibility of toxicity induction in 12-week-old mice through the administration of 12.5 nm colloidal AuNPs with regular shape for eight days, in doses of 40, 200, and 400 µg/kg/day. The evaluation of this parameter aimed to clarify the possibility of AuNP use in, e.g., drug delivery or disease diagnostics. Hematological analysis evaluating white and red blood cells, platelets, hemoglobin, and hematocrit was performed using a hematological analyzer (Coulter T540 hematology system). Histopathological evaluation was performed based on H&E (hematoxylin-eosin) staining. The distribution of gold nanoparticles was characterized using GF–AAS (graphite furnace atomic absorption spectrophotometry) and ICP–MS (inductively coupled plasma mass spectrometry). The concentration of gold nanoparticles in the liver, kidneys, spleen, and lungs increased with dosage. Considering different sizes of organs, the total percent of the applied dose detected was highest in the liver, followed by kidneys and the spleen. In turn, the level of AuNP in the blood was independent of the administered doses, which indicates that uptake and absorption of gold nanoparticles mainly occur in tissues. In addition, the percentage of accumulated gold decreases with the increase in AuNP dose, which suggests efficient removal of nanoparticles from the body.

Concentrations of urea nitrogen, uric acid, and creatinine were examined to evaluate AuNP nephrotoxicity. Moreover, total bilirubin and alkaline phosphatase levels in the blood were used for functional evaluation of the liver and bile ducts. The biochemical analysis of these parameters did not show statistically significant differences in any metabolites, regardless of the dose used. Furthermore, the hematological analysis did not show statistically significant differences between gold nanoparticle incubated mice and control samples. This confirms the conclusion that gold nanoparticles do not cause extensive inflammation in mice. In addition, for observation of possible toxic effects, macroscopic morphological analysis of tissues was carried out. Tissue damage was not shown in any of the sections taken from the kidneys, liver, spleen, brain, or lungs. Gold nanoparticles also did not affect the weight of mice regardless of the dose of nanoparticles used [96].

The effect of the surface functionalization of gold nanoparticles was studied by Zhang et al. (2020); AuNPs stabilized by PEG (polyethylene glycol) are commonly used as nanodrug carriers due to their biocompatibility, obtained through nanoparticle stabilization. The study compared surface functionalization of nanoparticles using PEG (AuPEG) and Trolox (AuTrolox). The second substance is a Vitamin E derivative, with its administration leading to inhibition of oxidative stress through the removal of ROS and nitrogen oxides.

The studies were conducted in vitro on the SH-SY5Y neuroblastoma cell line. The cells were incubated with gold nanoparticles of 4.5, 13, and 30 nm and 25 μg/mL for 24 h. An MTT test was used to investigate the toxicity of SH-SY5Y cells. Using a laser confocal microscope, the level of reactive oxygen species was measured. Furthermore, malondialdehyde (MDA, malondialdehyde) levels were assessed as a marker of oxidative stress. Subsequently, mitochondrial membrane potential was also investigated using JC-1 dye and confocal microscopy techniques. In turn, the effect of gold nanoparticles on apoptosis values was measured using a flow cytometer and a commercial reagent kit. To determine which apoptotic proteins are involved in signaling pathway induction, Bcl-2, caspase-3, and PARP (poly (ADP-ribose) polymerase) proteins were investigated. Finally, using the ICP–MS (inductively coupled plasma mass spectrometer) method, the distribution of AuPEG and AuTrolox in individual mouse organs was investigated.

AuPEG nanoparticles of 4.5 nm were shown to have higher toxicity than particles of other sizes. Therefore, they were selected for further studies evaluating the effects of antioxidants on nanoparticle-induced oxidative stress.

For AuPEG, six times higher levels of ROS and two times higher MDA values were demonstrated compared to control. However, a significant reduction in MDA and ROS occurred after the surface of nanoparticles was functionalized with Trolox. Furthermore, through increased green fluorescence signal and reduced red signal of JC-1 dye, mitochondrial damage in cells incubated with AuPEG was confirmed. A decrease in green fluorescence levels was also observed when AuTrolox was used.

AuPEG and AuTrolox nanoparticles (4.5 nm) were used to evaluate apoptosis, at concentrations of 2.5, 5, 10, 25, 50, 75, and 100 μg/mL, during 24-hour incubation. AuPEG was shown to significantly affect cell viability, with a decrease of almost 40% confirmed for concentrations of 50, 75, and 100 μg/mL. However, AuTrolox mediated inhibition of apoptosis was also detected. After 48-hour incubation of SH-SY5Y cells, at 25 μg/mL concentration, the apoptosis level in AuPEG treated cells was 40%, while it was 20% for AuTrolox. Flow cytometry results also confirmed differences in apoptosis induction. Furthermore, Western-blot analysis showed a decrease in Bcl-2 protein expression, and activation of caspase-3 and PARP.

The study confirmed that the use of Trolox on the surface of gold nanoparticles significantly reduces the adverse effects of ROS and MDA and improves antioxidant enzyme activities compared to AuPEG. According to the researchers, Trolox is an antioxidant with proven effects in the reduction of oxidative stress. The authors also state that combining antioxidants with gold nanoparticles can increase their activity.

The possibility of AuPEG induction of apoptosis via the mitochondrial apoptosis signaling due to ROS presence was also confirmed. However, this process was reversed after the application of AuTrolox.

An in vivo model of male mice weighing approximately 20 g was used to further assess the neurotoxicity of nanoparticles, with the animals receiving ranging NP doses (12.5 and 25 mg/Kg).

Using ICP–MS, the greatest accumulation of gold nanoparticles was detected in the liver and then in the spleen, especially compared to the heart, kidneys, and lungs. This occurrence was associated with increased phagocytic activity in the cells of the mononuclear phagocyte system. Furthermore, higher concentrations of nanoparticles translated into their higher accumulation in the test organs. No difference in body weight was observed between mice treated with AuPEG and AuTrolox.

**Figure 2:**
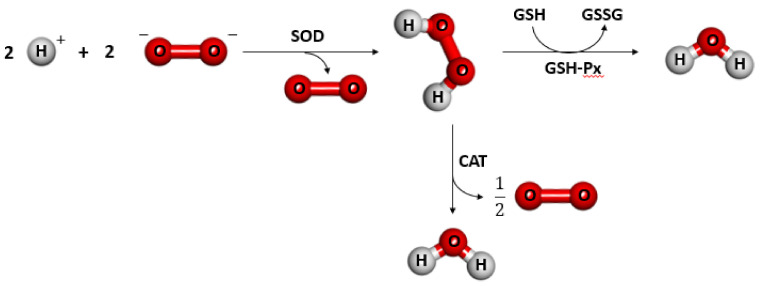
The principle of antioxidant action. SOD (superoxide dismutase), CAT (catalase), GSH-Px (glutathione peroxidase), GSH (reduced glutathione), GSSG (oxidized glutathione).

In samples taken from the mouse hippocampus, the levels of antioxidant enzymes such as SOD (superoxide dismutase), CAT (catalase), and GSH-Px (glutathione peroxidase) were evaluated. The principle of antioxidant action is presented in Figure 2.

Application of AuPEG at 25 mg/kg for three months caused a decrease in MDA and antioxidant enzyme levels (SOD, CAT, GSH-Px) in the hippocampus, compared to AuTrolox [72].

Through induction of oxidative stress in cells, gold nanoparticles lead to the accumulation of free radicals and thus reduce the activity of liver antioxidant enzymes (SOD, CAT) and GSH levels [97].

Overall, the publication of Zhang et al. (2020) demonstrates the possibility of inducing oxidative stress and apoptosis by 4.5 nm PEG-stabilized AuNPs. Inhibition of this process through the use of an antioxidant—Trolox—was also noted. This research provides a reasonable basis for the potential development of a nanoparticle drug delivery system [72].

A mouse fibroblast cell line (Balb/3T3) was the subject of research by Coradeghini et al. (2013). Gold nanoparticles of 5 and 15 nm in size and concentrations of 10, 50, 100, 200, and 300 μM were used. Incubation with AuNP was conducted for 2, 24, and 72 h. Culture performance tests assessing colony-forming efficiency (CFE) and Trypan blue were used to determine the toxicity of nanoparticles. A transmission electron microscope was also used to evaluate NP location qualitatively. In turn, the quantitative uptake of nanoparticles by Balb/3T3 cells was characterized using ICP–MS.

In the CFE test, smaller nanoparticles showed higher toxicity at 72 h of incubation, especially at concentrations above 50 μM. For 15 nm NPs, no statistically significant differences were observed for any concentration and incubation time. Based on the Trypan blue dye test, 5 and 15 nm nanoparticles were not toxic to mouse fibroblasts, regardless of the concentration and exposure period used.

Furthermore, the cells were incubated with 10 and 30 μM gold nanoparticles for 2 and 24 h. Using a transmission electron microscope, cellular internalization was determined to occur in both AuNP sizes used. Moreover, enclosure of nanoparticles inside vesicles was observed. However, AuNPs did migrate to other organelles, such as the nucleus, mitochondria, or Golgi apparatus. The number of nanoparticles collected in endocytotic vesicles increased with concentration. Moreover, autophagosome formation was also shown to be possible. Furthermore, it was noted that even after 2 h of incubation, the nanoparticles already reached the the end of the endo/lysosomal pathway.

The uptake of nanoparticles after 2 h was confirmed using ICP–MS (plasma mass spectrometry). The number of nanoparticles increased with incubation time and was higher in those 15 nm in size.

Summarizing, the study confirmed 5 nm AuNP toxicity at incubation time 72 h and concentrations above 50 μM. Therefore, the importance of the nanoparticle size used should be taken into account in studies determining their effects on cell biology [98].

Scientists in Gdansk 2019 investigated the effect of the shape of gold nanoparticles on toxicity in cancer cells. They used four cell lines: human fetal osteoblast (hFOB 1.19), human bone osteosarcoma (143B), human osteosarcoma cell line (MG-63), and pancreatic duct (hTERT-HPNE). The gold nanoparticle shapes investigated were nanosphere, nanostar, and nanorod. Nanoparticle morphology was characterized using SEM and TEM microscopes. The average length of nanoparticles of a nanosphere shape was 14 nm, while the size of the nanostars was about 200 nm. Nanorods had an average length of 45 nm and a diameter of 16 nm. The cell lines investigated were incubated with different AuNP shapes for 24 h.

Cell viability was tested using the MTT test to measure the cellular activity of NADPH-dependent oxidoreductase, as reduced cell life span may be associated with the process of apoptosis. In order to assess the ability of live undamaged cells to collect dye in lysosomes, neutral red (NR) assay was used, as this test also allows for measuring the integrity of the cell membrane. Concentrations of nanoparticles of 0.3, 0.6, 1.2, 2.5, and 5 μg/mL were used for both tests.

A comparison of tests determining cell viability showed that nanostars had the most significant impact on reducing cell lifespan. In this shape, the survival rate of the cell lines investigated decreased as the concentration increased. The highest susceptibility to toxicity caused by nanostars was attributed to the 143B cell line cells. However, the NR test did not show similar activity for nanostars at 0.3 μg/mL concentration. Nanorods showed significant toxicity at higher concentrations (2.5 and 5 μg/mL), especially with MG63 and 143B cell lines. However, the NR test results showed a lower effect on cell survival at the same concentrations of these nanoparticles. Nanospheres exerted the smallest impact on cell line survival. Despite that, a slight reduction in cell life of 143B cells was shown in the MTT test. Moreover, the results of both tests point to toxicity mediated by AuNPs via alterations in mitochondrial activity (MTT) and integrity of the cellular membrane (NR).

Owing to these toxicity results, nanostars and nanorods were further analyzed in relation to their effect on the levels of apoptotic proteins. The NR test showed that hFOB1.19 cells were the most resistant to nanoparticles, prompting the use of 2 remaining cell lines (MG63 and 143B) for further study. The concentrations of a proapoptotic protein (Bax) and antiapoptotic protein (Bcl-2) were determined using the Western blot method. The concentrations used were 1 and 2 μg/mL for nanorods and 0.1, 0.3, 0.6, and 1 μg/mL for nanostars. Both 143B and MG63 cell lines showed an increase in proapoptotic protein levels in the presence of nanorods. Concerning the antiapoptotic protein, a decrease in Bcl-2 levels was observed only in MG63 cells. For the 143B cell line, an increase in Bcl-2 at 1 μg/mL and a decrease at 2 μg/mL was demonstrated.

In turn, nanostars increased Bax levels as their concentrations increased for both cell lines. They also caused a decrease in Bcl-2 expression in both 143B and MG63 cells. The most significant changes in Bax and Bcl-2 protein levels were observed at AuNP concentration of 1 μg/mL.

The morphology of the hTERT-HPNE cell line was analyzed in a TEM. Nanostars and nanorods were shown to penetrate cells and cause changes in their ultrastructure. Nanostars were used at a concentration of 10 μg/mL, resulting in intensive vacuolization of the cytoplasm, with an especially prominent appearance of autophagic vacuoles. At a concentration of 50 μg/mL, cell damage occurred, consisting of cell membrane rupture, cytoplasmatic vacuolization, and cell degeneration.

In turn, nanorods at a concentration of 10 μg/mL localized outside the cell, along the cell membrane. As a result of endocytosis, they were also observed in endosomes. At this concentration, the cell showed an unchanged structure of the rough endoplasmic reticulum, and the presence of numerous autophagosomes. At a higher concentration (50 μg/mL), cell degradation occurred, including cell membrane damage,.

Given the demonstrated relationship between the shape of the nanoparticles used and the resulting toxicity, this parameter should be considered when designing biomedical applications [99].

Summarizing, the size of the gold nanoparticles used is essential for their biokinetics. Smaller nanoparticles (approximately 10 nm) accumulate in many organs, e.g., liver, spleen, kidneys, testicles, and lungs, and blood [66]. Smaller nanoparticles <10 nm show more significant toxicity compared to the larger ones, probably due to their ability to penetrate the cell nucleus [68]. However, renal filtration is disturbed for larger gold nanoparticles (>65 nm), resulting in a lack of urine excretion. Instead, they are eliminated from the blood by the reticuloendothelial system and tend to accumulate in the spleen and liver [66].

### 5.3. Comparison of Silver and Gold Nanoparticle Toxicity

Several research groups evaluated silver (dispersion NP) and gold nanoparticle (colloidal solution) toxicity in comparative experiments. Results of one of such study conducted on a mouse model were published by Shrivastwa et al. in 2015. The study was based on male Swiss albino mice, 25–30 g in weight. Blood and tissues from the brain, liver, kidneys, and spleen were used for the experiment. The nanoparticles analyzed were silver and gold, with a size of 20 nm, at concentrations of 1 and 2 μM/kg. AgNP and AuNP were administered to mice interally for 14 days.

The amount of reactive oxygen species in treated mice was examined through the analysis of the fluorescence level of the DCFH-DA dye in blood and mouse tissues. GPx (glutathione peroxidase) and GST (glutathione-S-transferase) were used to assess the level of antioxidant enzymes in the blood and tissues. Within the tissues, the ratio of GSH: GSSG was evaluated (reduced glutathione: oxidized glutathione), while total glutathione levels were analyzed in blood. Furthermore, the level of inflammation was marked in soft tissues using IL-6 (interleukin-6).

Concerning the results of the analysis, significant weight loss was observed in mice exposed to nanoparticles, especially at a higher dose (2 μM). Moreover, AgNP seemed to cause more substantial weight loss than AuNP. The intensity of fluorescence increased after incubation of blood with nanoparticles in both doses compared to control, with the largest difference noted for AgNP at 2 μM dose. Concerning blood analysis, AgNPs at a dose of 2 μM were found to be the most stimulating, as AgNPs inhibited analyzed enzymes to a greater extent than AuNPs.

The effect of gold and silver nanoparticles on soft tissues is presented in Table 4.

GPx (glutathione peroxidase) activity decreased in the brain, especially at a concentration of 2 μM AgNP. In contrast, its level increased significantly in the kidneys at the same dose. An increase in value was also observed at a concentration of 1 μM for both types of NP. In the liver, gold had a stimulating effect on GPx levels, especially at a dose of 1 μM, while silver inhibited this enzyme at the same amount.

Concerning GST levels in the brain, one µM AgNP resulted in the most significant increase of this protein. In the liver and kidneys, all NPs acted inhibitory, with the most considerable effect attributed to AgNPs at 2 µM. In turn, in the spleen, the largest inhibitory potential was presented by 2 µM AgNPs.

An increase in the fluorescence intensity of the DCFH-DA dye was noted in blood and all soft tissues incubated with AgNPs and AuNPs. In the brain, liver, kidneys, and spleen, 2 μM levels had the most promoting effects, especially concerning silver nanoparticles. Furthermore, a significant increase in IL-6 levels was observed in both types of nanoparticles, especially at a dose of 2 μM, compared to the control sample.

The application of nanoparticles also significantly affected toxicity. This process manifested through inflammation and increased ROS release resulting from oxidative stress. More pronounced adverse effects were attributed to silver nanoparticles, especially at higher analyzed doses [75].

In another study, Barkur et al. (2020) studied oxidative stress caused by silver and gold nanoparticles on human red blood cells (RBC) using Raman spectroscopy. The cells were incubated for 24 and 48 h with 50 nm silver and gold nanoparticles. Moreover, thiol levels were studied through absorbance measurements.

Raman spectroscopy showed an adverse effect of increasing concentrations of nanoparticles on the binding of oxygen to hemoglobin. After 24 h of incubation, minimal spectral changes were observed. RBC treated with 100 μL of silver nanoparticles ruptured after 48 h of incubation, making it impossible to perform spectroscopy. Using 50 μL of NPs, more significant spectral fluctuations were shown in cells incubated with silver than gold nanoparticles. The Raman spectra showed more variability with AgNPs of 30 nm, compared to larger sizes (50, 80 and 100 nm). In addition, for AuNPs, the highest and lowest spectral variability levels appeared at 30 and 10 nm sizes, respectively. This indicates a more harmful effect of AgNPs on the affinity of hemoglobin in erythrocytes for binding oxygen. Due to their antioxidant properties, examination of thiol levels in cells allowed for evaluation of oxidative stress in the presence of nanoparticles. Significant changes in thiol values indicate that such a state can result from NP administration, with more reduced thiol content observed for silver nanoparticles, indicating greater levels of oxidative stress.

In addition, changes in hemoglobin structure resulting from incubation with NPs were demonstrated. This occurrence was related to the possibility of nanoparticle adherence to the cell membrane of erythrocytes, causing oxidative stress. Adverse effects of nanoparticles on hemoglobin oxygen-binding ability were also observed. Such processes could potentially cause negative side effects of metal nanoparticle penetration into the human body [100].

**Figure 3:**
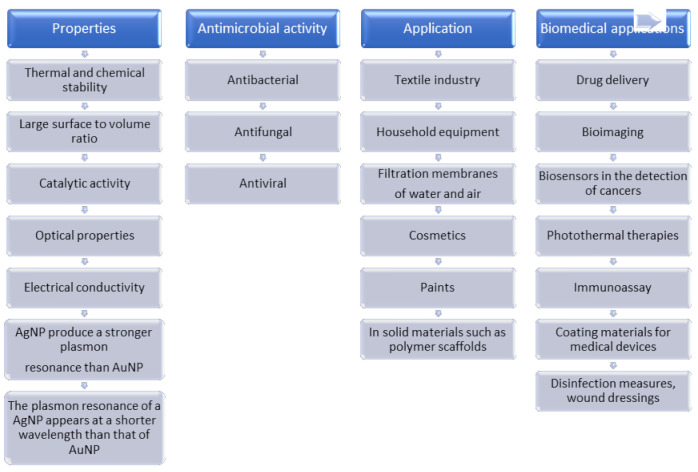
Main properties and applications of silver and gold nanoparticles.

The main properties and applications of silver and gold nanoparticles are shown in Figure 3.

The antimicrobial activity of nanoparticles is related to their electrostatic interaction with negatively charged cell surfaces, which improves their ability to penetrate cellular membranes. This process leads to the damage of biological membranes, coagulation of proteins, stimulation of ROS production, and ultimately to the reduction of microbial viability [10,23,101,102,103].

The antimicrobial activity of silver nanoparticles occurs through their attachment to the cell wall and penetration to the cytoplasm. There, the released silver ions interact with proteins at the amino acid level, disrupt electron transport in the respiratory chain and inhibit DNA replication. Due to the release of silver ions and alteration in the cell surface structure, cellular enzymes are deactivated, resulting in ROS production. These changes induce toxicity and ultimately lead to microbial death [10,23,30,101,102]. In turn, the antifungal activity of AgNPs is associated with disruption of membrane potential due to interaction between AgNPs and cell membrane of fungi, e.g., *Candida albicans, Trichophyton rubrum, Stachybotrys chartarum,* and *Mortierella alpina*. AgNPs induce the formation of perforations on the cell membrane surface, leading to osmotic shock and ultimately fungal death [101]. Finally, the antiviral activity of AgNPs occurs via the inhibition of virus attachment to the cell surface. Nanoparticles cause denaturation of disulfide bridge, affecting associated modifications of viral proteins [101].

The antimicrobial effect of gold nanoparticles is related to their attachment to the bacterial cell wall, resulting in the creation of pores and penetration into the intracellular space. There, they disrupt the metabolic processes, bind to DNA and inhibit the transcription process, and distort the ribosome units for tRNA binding. These effects ultimately result in a breakdown in the biotic mechanism of the bacteria [10,23,101,102]. Antifungal activity of gold nanoparticles has been confirmed in, among others, *Candida albicans* [101,103]. Furthermore, AuNPs also demonstrated antiviral activity by inactivating the virus via destruction of its capsid and inhibition of viral entry into cells [101].

Antimicrobial activity also depends on the nanoparticle size, shape, concentration, and surface modification [10,23,30,101,102,103].

The large surface-to-volume ratio of silver and gold nanoparticles enables high absorption of various molecules, e.g., polymers and therapeutic agents, facilitating their common use in biomedical fields [10,102]. Furthermore, a large surface area also improves the interaction of nanoparticles with bacterial cells [23].

The unique optical properties of gold and silver nanoparticles occur due to the excitation of localized resonance of surface plasmons, with greater excitation occurring on silver NPs, compared to their gold counterparts [10,102,104].

## 6. The Blood–Brain Barrier

Due to the increasing use of nanoparticles, the possibility of them crossing the blood–brain barrier (BBB) remains a significant concern. This process was confirmed, e.g., by Tang et al., in an experiment based on rat brain microvessel vascular endothelial cells (BMVEC) and astrocytes (AC) incubated with spherical AgNP (100 µg/mL). Under culture conditions, nanoparticles were observed to localize inside endothelial cells. NPs were described to enter the cells to transcytosis, a process that also allows them to infiltrate other tissues of the organism. Further in vitro studies have also confirmed the possibility of AgNPs crossing the blood–brain barrier and have identified potentially harmful effects of AgNP on brain tissue and their interaction with cellular organelles such as mitochondria and the rough endoplasmic reticulum [12,105].

Hence, due to their ability to cross the BBB, spherical AgNPs are considered as a potential neurotoxin. The increased transport of fluorescein by BBB indicates an NP size-dependent increase in BBB permeability, correlated with the severity of immunotoxicity. AgNPs have been shown to induce an increased release of pro-inflammable mediators, i.e., tumor necrosis factor (TNF-α), interleukins (e.g., IL-1β), and prostaglandin E2 (PGE2) in concentration of AgNP 50 µg/cm^3^, which are associated with increased rBMEC (rat brain microvascular endothelial cells) monolayer permeability and may cause BBB dysfunction. The permeability of primary rBMEC monolayers was evaluated as the percentage of fluorescein flow across the rBMEC monolayers. AgNP was used at a concentration of 15 µg/cm^3^. Furthermore, systemic exposure to AgNPs, depending on their size, can result in microvascular damage to the brain. It has been reported that smaller (25 nm) AgNPs produce a more robust inflammatory response, correlated with increased brain microvascular permeability and cell monolayer perforation compared to larger AgNPs (40 and 80 nm) [88].

Feeding mice with 34 nm silver nanoparticles for two months significantly increased the concentration of AgNP in the brain compared to one-day incubation. The effect of month-long distilled water administration on the AgNP concentration was also investigated, proving that the level of nanoparticles remained at about 6%. This may be related to the mechanism of endocytosis and exocytosis across the blood–brain barrier and confirm the accumulation of silver nanoparticles in the brain. Therefore, low levels of AgNP removal from the brain may be a risk to patients treated with products containing NPs, potentially resulting in an opposite rather than expected effect during long-term use [94,106].

Moreover, an experiment studying the effects of long-term oral administration of AgNPs confirmed continuous accumulation of silver nanoparticles in the mouse brain due to exposures lasting up to 4 months. The accumulation of 34 ± 1.4 nm AgNPs at a concentration of 25 μg/mL in the brains of experimental animals was significantly higher after 4-month vs. 2-month administration. Slow removal of AgNP from the brain was also observed after discontinuation of NP intake. After specific administration periods, the amount of silver accumulated in brain tissues was determined by neutron activation analysis (NAA) and compared with Morris water maze (MWM) behavioral test results. The presence or absence of AgNP in the body did not have an apparent effect on memory: differences in dynamics and ranges of parameters described above were found in both the experimental and control subgroups. However, there is a possibility of the results being affected by the memory effect, as the animals, after testing in MWM, could have retained information about the research area, such as the structure of its internal space and the location of external clues [107].

Furthermore, Lasagna-Reeves et al. (2010) confirmed the ability of 12.5 nm colloidal AgNPs with regular shape for crossing the blood–brain barrier in mice. Given the relatively constant level of gold in the blood after AgNP administration at different doses, increased accumulation of gold in the brain suggests their uptake from the blood to the brain. An increase of AuNP accumulation with dosage confirms the possibility of nanoparticle use for targeted therapy in the brain without production of detectable toxicity. These properties emphasize the potential application of gold nanoparticles to treat and diagnose neurodegenerative disorders [96].

Nanoparticles in the brain were also observed in mice administered 4.5 nm gold nanoparticles intravenously for three months. Nonetheless, AuPEG accumulation in the brain was much higher than with AuTrolox. Furthermore, morphological changes have been studied through HE staining. In the AuPEG group, some hippocampus neurons were condensed and darkly stained in the CA1, CA3, and Hilar regions, indicating their damage and loss. However, cells incubated with AuTrolox were rarely stained and had visible nuclei, implying a Trolox mediated decrease in AgNP toxicity.

Next, immunohistochemical staining was performed using a primary antibody against NeuN. This protein is localized in nuclei and nuclear cytoplasm of most central nervous system neurons in mammals and is considered a reliable indicator of post-mitotic neurons. The intensity of hematoxyline staining was studied using a tissue cytometer (TissueFAXS-plus), showing decreased CA1, CA3, and Hilus regions at both 12.5 and 25 mg/kg AuNPs. With the addition of Trolox, an increase in NeuN antibody expression and inhibition of apoptosis were observed when compared to the AuPEG group.

After isolating and homogenizing the hippocampus, LDH (lactate dehydrogenase) levels were investigated. After treatment of cells with AuPEG at a 25 mg/kg concentration, a two-fold increase in LDH levels was observed, indicating that AuPEG may cause cell death [72].

## 7. Conclusions

The history of the use of silver and gold nanoparticles began in antiquity. Ancient Greeks used silver-coated dishes to store wine for longer times [108]; this metal was also used to clean wounds and treat infections [109]. In addition, gold was used for medical treatment, e.g., smallpox, skin ulcers, and measles [108].

Due to the development of nanotechnology and the use of nanoparticles, it is necessary to study their effects on cells. Many parameters of NPs influence toxicity, including size, shape, concentration, type of coating, and incubation time [33,106]. It is worth noting that response to NPs also depends on the cell type. Hence, to increase the safety of their use, this aspect needs to be considered, especially in the context of biomedical applications [106]. Differences in physicochemical variables make the assessment of nanoparticle toxicity a relatively complex process. Even minor modifications to the surface coating can affect biodistribution in the body, e.g., through differences in the uptake of nanoparticles by macrophages, the level of accumulation in different organs, or the rate of removal from the organism [33,110].

The effect of an increase in the concentration of silver nanoparticles leading to apoptosis is shown in Figure 2. AgNP toxicity is most likely associated with their surface oxidation and the release of silver ions, which cause biochemical changes, abnormalities in cell functions, and neurotoxic modifications [106,111]. The nanotoxicity mechanism relies on the production of ROS, including singlet oxygen, superoxide radical ions, oxide radicals, superoxide ions, hydrogen peroxide, and hydroxyl radicals. ROS generation processes involve mitochondrial respiration and the subsequent release of ROS into the cytoplasm through pores in mitochondrial membranes formed by nanoparticles. In normal cells, a balance is maintained between intracellular antioxidants and ROS. However, nanoparticles can directly damage the mitochondria, causing an increase in intracellular ROS, which may stimulate the further release of ROS from mitochondria in a process known as ROS-induced ROS release. This process can significantly increase intracellular ROS levels and exacerbate oxidative imbalances. High levels of ROS can result in oxidative stress and damage to cellular organelles, DNA, cell membranes, ion channels, and cell surface receptors, leading to toxicity [33,112].

AgNPs have a higher potential for toxicity compared to AuNPs. Therefore, an increase in the share of the use of AuNP was observed, especially in functionalized, therapeutic, and diagnostic methods [106]. Given the primary use of AgNP in clothing and skin surface disinfectants, the retention of silver in the stratum corneum due to aggregation can be beneficial. It could be considered a reservoir of silver ions that may promote and prolong the antibacterial effect. Since the aggregates are several μm in size, they will not penetrate deeper skin layers and will eventually be removed from the stratum corneum by exfoliation. Hence, the formation of aggregates can be seen as a mechanism of detoxification due to the fact that only a rudimental amount of silver reaches the systemic circulation [62].

Gold nanoparticles, characterized by increased biocompatibility, stability, and low toxicity, are considered one of the most suitable carrier systems for medicines. AuNPs functionalized by PEG, due to their ability to bind to cell membranes, have an increased ability to penetrate target cells. Furthermore, fluorescent dye coating allows for monitoring of their movement [113].

Increased use of hazardous chemicals used in the production of nanoparticles could become a serious cause of environmental degradation. Hence, the use of nanoparticles of plant origin could aid in the reduction of harmful chemicals in NP synthesis, as research indicates the possibility of their therapeutic use. They were described to manifest anticancer potential in the treatment of lung, liver, and cervical cancer, and were proposed for use in antidiabetic drugs, acting as an inhibitor of α-amylase. The green synthesized nanoparticles were proven to prevent the development of microbes and have therefore been used in disinfectants and as an antimicrobial coating on medical devices such as catheters. However, the mechanisms of action of these nanoparticles are not yet fully understood. While additional research is needed, the results of past experiments provide an optimistic perspective of a potential increase in the use of plant-based nanoparticles in industry and medicine [109,114].

The emerging discrepancies between literature data on the correlation between the physicochemical properties of nanoparticles such as size, shape, type of functionalized surface, and induced toxicity are the inspiration for further scientific research [111]. It is also essential to more broadly investigate the correlation between the physicochemical properties of nanoparticles and their biodistribution in the organism, as it could help assess the risk of their use in humans [115].

## Figures and Tables

**Table 1:** Summary of the effects of silver nanoparticles on different types of cell lines: normal human lung fibroblast cells (IMR-90); pulmonary epithelial (A549), renal epithelial (A498), neuronal (Neuro 2A) cell lines; human Chang liver cells; human glioblastoma cells (U251); human hepatocellular carcinoma cells (HepG2); human fibrosarcoma (HT-1080), human skin carcinoma (A431); human lung carcinoma epithelial-like cell line (A549); Chinese hamster ovary cells (CHO); macrophage (RAW 264.7, J774.1) cell lines; mouse peritoneal macrophage cell line (RAW264.7); primary bovine retinal endothelial cells (BREC); immortalized human pancreas duct epithelial cell line hTERT-HPNE (CRL-4023); human pancreatic ductal carcinoma PANC-1 (CRL-1469); human colonic epithelial cell line (HT29); human stomach cancer cells (SGC-7901), and human breast adenocarcinoma cells lines (MCF-7).

Tested Nanoparticle Parameter	Type of Cells			Effect
	Normal Human Cells	Human Tumor Cells	Animal Cells	
Increase in AgNP concentration	IMR-90 [76];	U251 [76];	CHO [83];	Decrease of cell viability and increased apoptosis, increased ROS production
	A549, A498, Neuro 2A [77];	Hep G2 [77,80];	RAW 264.7, J774.1 [77];	
	human Chang liver cells [78];	HT-1080, A431 [81];	RAW264.7 [84];	
	CRL-4023 [79];	A549 [82];	BREC [85];	
		CRL-1469 [79];		
Smaller AgNP size	IMR-90 [75];	human glioblastoma cells [76];	BREC [85]	Increased toxicity
	lymphocyte cell [86];	A549, SGC-7901, HepG2, MCF-7 [87];	Primary rat brain microvessel endothelial cells [88]	
	rat alveolar macrophages [89];			
	HT29 [73];			

**Table 2:** Effect of gold nanoparticle size on hematology results in mice incubated with AuNP at a dose of 4000 μg/kg for 28 days. Numerical values are given in nm ↑↑—largest increase, ↑— increase ↓— decrease.

Leukocytes				Erythrocytes				Hemoglobin and Mean Corpuscular Hemoglobin Concentration			
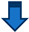 5	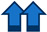 10	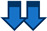 30	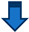 60	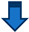 5	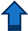 10	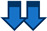 30	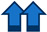 60	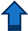 5	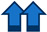 10	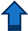 30	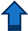 60
**Hematocrit**				**Mean erythrocyte volume**				**Thrombocytes**			
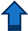 5	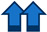 10	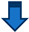 30	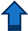 60	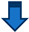 5	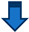 10	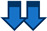 30	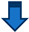 60	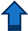 5	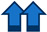 10	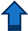 30	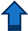 60

**Table 3:** Levels of biochemical enzymes in the blood of mice treated with gold nanoparticles for 28 days at a dose of 4000 μg/kg. ALT—alanine transaminase, AST—aspartate transaminase, GLOB—globulin, CREA—creatinine, ALB—albumin, TBIL—total bilirubin. ↑↑—largest increase, ↑—increase, decrease, ↓↓—largest decrease.

ALT				AST				GLOB			
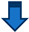 5	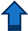 10	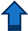 30	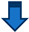 60	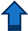 5	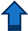 10	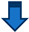 30	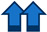 60	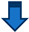 5	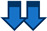 10	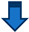 30	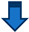 60
**CREA**				**ALB**				**TBIL**			
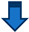 5	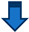 10	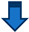 30	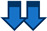 60	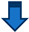 5	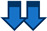 10	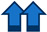 30	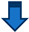 60	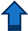 5	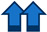 10	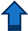 30	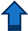 60

**Table 4:** The effect of gold and silver nanoparticles on soft tissues. GSH: GSSG—reduced glutathione: oxidized glutathione; GPx (glutathione peroxidase); ROS—reactive oxygen species; AuNP—gold nanoparticles; AgNP—silver nanoparticles; ↓—decrease ↑increase of fluorescence.

	GSH: GSSG		GPx		GST		ROS	
	AuNP	AgNP	AuNP	AgNP	AuNP	AgNP	AuNP	AgNP
**Brain**	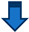	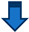	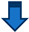	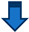	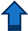	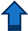	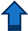	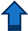
**Liver**	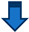	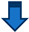	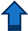	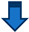	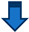	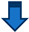	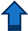	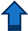
**Kidneys**	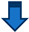	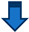	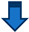	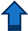	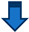	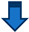	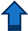	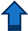
**Spleen**	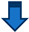	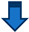	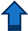	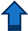	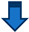	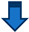	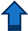	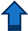

## Data Availability

Not applicable.
